# Targeted Lipidomics and Transcriptomics Reveal Key Nutrients Regulating Ovarian Development in *Coreius guichenoti*

**DOI:** 10.3390/ani16162602

**Published:** 2026-08-20

**Authors:** Jian Zhu, Yihao Xiao, Haiyue Tan, Maohua Li, Xin Lu, Xiaowen Gao, Yongjun Chen, Huantao Qu

**Affiliations:** 1 Chinese Sturgeon Research Institute, China Three Gorges Corporation, Yichang 443100, China; zhu_jian3@ctg.com.cn (J.Z.); limaohuayu@foxmail.com (M.L.); gxw_as@163.com (X.G.); 2 Hubei Key Laboratory of Three Gorges Project for Conservation of Fishes, Yichang 443100, China; 3 Institute of Three Gorges Ecological Fisheries of Chongqing, College of Fisheries, Southwest University, Chongqing 400715, China; xtryst111@163.com (Y.X.); hyyy0712@163.com (H.T.); 18296879796@163.com (X.L.)

**Keywords:** *C. guichenoti*, multi-omics, ovarian development, fatty acids, vitamins

## Abstract

In this study, targeted metabolomics and transcriptomics were applied for the first time to compare fatty acid contents, vitamin levels, and gene expression profiles between Stage II and Stage IV ovaries of LBG. The data revealed a rise in total SFA and PUFA as the ovary progressed from Stage II to Stage IV. The marked increase in n-3 PUFA from 11.5% in Stage II to 26.8% in Stage IV ovaries was primarily attributed to DHA. Despite a reduction in n-6 PUFA, ARA and its metabolism-related fatty acids all increased, suggesting indispensable roles for both ARA and DHA in the vitellogenesis of LBG. Consistent with the changes in fatty acid profiles during ovarian maturation, transcriptome analysis revealed that many upregulated differentially expressed genes (DEGs) showed significant enrichment in fatty acid biosynthesis, arachidonic acid metabolism, and steroid hormone synthesis pathways. The concentrations of VC and VE increased, whereas VA declined during ovarian development from Stage II to Stage IV. Accordingly, many upregulated DEGs exhibited significant enrichment in ascorbate and aldarate metabolism, as well as vitamin digestion and absorption pathways. In addition to VC and VE, our transcriptome data also highlighted the importance of several B vitamins in oocyte development of LBG, warranting further investigation. Overall, these findings suggest that DHA, ARA, VC, and VE are key nutrients governing oocyte maturation in LBG. This study provides a solid theoretical basis for developing broodstock nutritional enhancement strategies in the artificial propagation of LBG.

## Simple Summary

Largemouth bronze gudgeon (*Coreius guichenoti*, LBG) is a rare and economically valuable fish species endemic to the upper reaches of the Yangtze River. The developmental arrest of ovaries from Stage II to Stage IV represents a major bottleneck in its artificial propagation, and nutrient fortification is considered an effective strategy to overcome this issue. In this study, we compared the differences in the amounts of fatty acids and vitamins, as well as gene expression profiles, between Stage II and Stage IV ovaries of LGB. The pronounced increases in total saturated fatty acids (SFA) and polyunsaturated fatty acids (PUFA) reflect the physiological demands of energy storage and structural construction during late ovarian development. The increase in total n-3 PUFA was driven by docosahexaenoic acid (DHA), while arachidonic acid (ARA) and its metabolism-related fatty acids all increased despite a decrease in total n-6 PUFA. In addition to DHA and ARA, vitamin C (VC) and VE were also identified as key nutrients regulating ovarian maturation in LGB based on targeted metabolomics and transcriptomics. These results establish a solid theoretical foundation for quantitatively determining the dietary requirements of these nutrients for captive LBG broodstock.

## 1. Introduction

Largemouth bronze gudgeon (*Coreius guichenoti*, LBG) is a rare endemic fish of the upper Yangtze River, valued for both its ecological significance and economic importance. Wild populations have declined sharply due to hydropower development, habitat fragmentation, and other anthropogenic disturbances, with some local stocks nearing depletion [1,2,3]. Artificial propagation and stock enhancement are critical for restoring wild populations, yet successful large-scale propagation depends on adequate broodstock reserves [4]. As wild capture for broodstock supplementation is no longer viable, captive rearing of reserve broodstock has become extremely urgent [5,6]. However, current rearing techniques remain suboptimal, with low maturation rates, unstable interannual reproductive performance, and poor fecundity severely limiting the efficient utilization of farmed broodstock [1,4]. In this context, elucidating the nutritional requirements for ovarian development in LBG is a critical step toward overcoming this artificial propagation bottleneck.

Ovarian development in fish proceeds through successive stages (oogonia proliferation, yolk accumulation, and oocyte maturation) and is highly sensitive to nutrient availability under captive conditions [7]. During ovarian development, fatty acids are typically mobilized from storage tissues (e.g., liver) to ovaries and oocytes, though the dynamics of this process vary among fish species [8]. For instance, hormonally mature European eel (*Anguilla anguilla*) exhibits distinct lipid redistribution patterns between muscle and ovary [9]. In flathead gray mullet (*Mugil cephalus*), muscle lipid content remains low and constant throughout ovarian development, whereas hepatic lipid content is relatively high and the hepatosomatic index increases with gonadal progression [10]. Nutrient reserves critically influence broodstock fecundity and offspring quality, with dietary nutrition playing a key role in enhancing reproductive performance [11]. Fatty acids and vitamins play indispensable roles in fish gonadal development among dietary components [12,13]. For example, dietary supplementation of broodstock with ARA enhances sperm motility, female fertility, and offspring fitness and survival in the medaka *Oryzias latipes* [14]. Dehydroascorbic acid (DHA) promotes oocyte maturation and quality in zebrafish by promoting pregnenolone production [15]. Vitamin E (VE) serves as a powerful antioxidant, shielding eggs from oxidative damage and thus enhancing egg quality and fertilization rates in Arabian yellowfin seabream *Acanthopagrus arabicus* [16]. However, in LBG, the categories and quantities of key nutrients that regulate broodstock reproductive performance remain unclear.

In LBG, a critical bottleneck occurs during the transition from Stage II (previtellogenic) to Stage IV (late vitellogenic), often resulting in developmental arrest that prevents oocytes from completing yolk deposition and final maturation. However, conventional nutritional approaches cannot effectively compare vitamin and fatty acid profiles across ovarian stages due to the scarcity of valuable biological samples. Omics techniques—such as metabolomics and transcriptomics—offer a viable alternative, requiring only minimal sample amounts. Metabolomics enables accurate quantification of key metabolites involved in ovarian development [17], while transcriptomic approaches provide insights into the molecular mechanisms underlying gonadal development in broodstock [18]. In this study, an integrated analysis of transcriptomics and targeted metabolomics was performed to systematically identify critical metabolic pathways and nutrients linked to the progression from ovarian Stage II to Stage IV. The findings will establish a solid theoretical foundation for quantitatively determining the nutritional requirements of captive LBG broodstock.

## 2. Materials and Methods

### 2.1. Rearing Conditions

All experimental fish were sourced from the Rare and Endemic Fish Propagation and Release Station, which belongs to the Xiluodu and Xiangjiaba Hydropower Stations along the Jinsha River in Yibin, China. LBG were reared in indoor circular concrete ponds (diameter 4 m, water depth 0.7 m) connected to a flow-through aquaculture system, with a daily water exchange rate of approximately 34.7 L/min. Water quality was maintained under optimal conditions: temperature 22.8–23.7 °C, pH 7.2–7.6, dissolved oxygen > 7.5 mg/L, and total ammonia nitrogen < 0.1 mg/L. The fish were maintained in complete darkness throughout the experimental period, except for a half-hour feeding session with dim light. They were fed once daily at 9:00 to apparent satiation with a commercial standard diet formulated for longsnout catfish *Leiocassis longirostris* (Chengdu Tongwei Animal Nutrition Technology Co., Ltd., Chengdu, China).

### 2.2. Sampling

**Figure 1:**
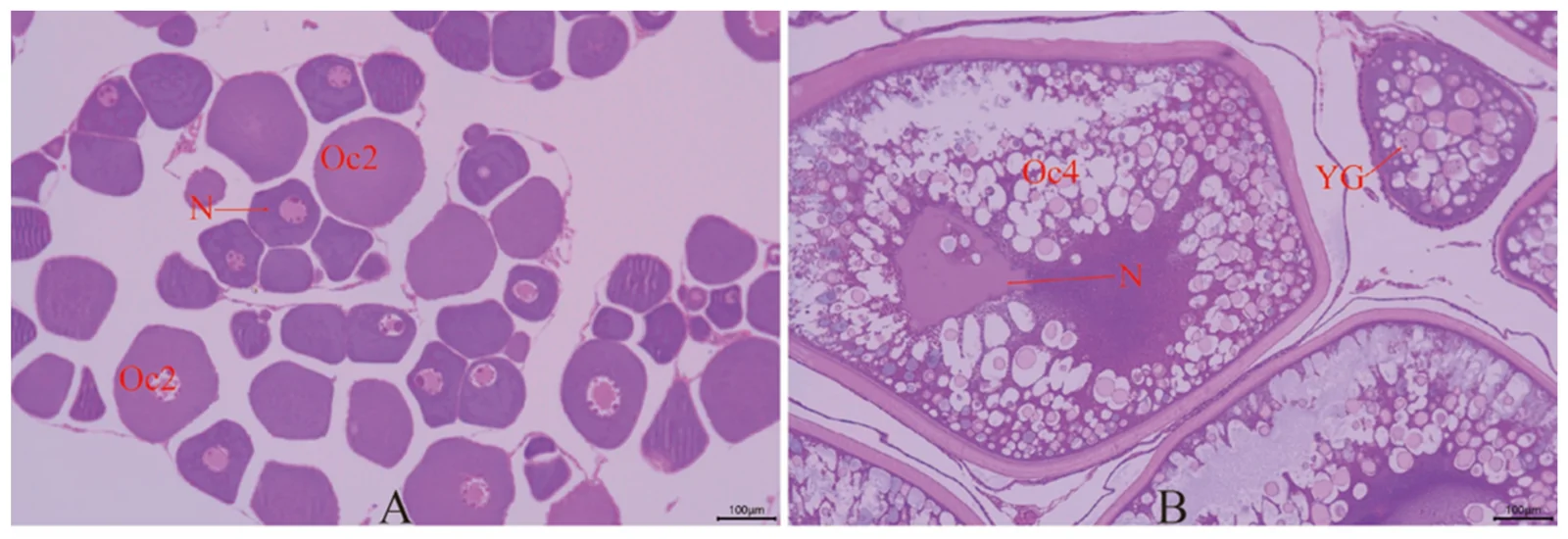
Histological appearance of Stages II and IV ovaries in largemouth bronze gudgeon (*C. guichenoti*). (**A**) Stage II ovary. (**B**) Stage IV ovary. N, nucleus. Oc2, Stage II oocyte. Oc4, Stage IV oocyte; YG, yolk granule.

Due to the scarcity of LBG, three individuals at Stage II and three at Stage IV were selected for ovarian sampling, designated as treatments G2 and G4, respectively. At sampling, body weight, body length, visceral mass, and ovary weight were recorded. Ovarian tissues were collected from both the left and right ovaries of each individual, which exhibited synchronous development and equivalent maturation status. To confirm the developmental stage (Figure 1), a portion of each sample was fixed in 4% paraformaldehyde solution for histological sectioning following Huang et al. [19]. The remaining ovarian tissue from each individual was fully mixed, flash-frozen in liquid nitrogen, and stored at −80 °C for subsequent omics analysis and quantitative real-time PCR (qPCR) validation. An additional three fish per stage were sampled for whole-body proximate composition analysis, and the percentages of moisture, crude protein, crude lipid, and ash were determined following the protocols of the AOAC [20].

### 2.3. Targeted Metabolomics Analysis

Ovarian samples were transported on dry ice to Biomarker Technologies Co., Ltd. (Beijing, China) for the analysis of fatty acids and fat-soluble vitamins (A, D, E, and K) by gas chromatography–mass spectrometry (GC-MS). Briefly, samples were mixed with extraction solution (isopropanol/n-hexane = 2:3, *v*/*v*, containing 0.2 mg/L internal standard) and steel beads, then vortexed, ground at 40 Hz for 4 min, and ultrasonicated in an ice bath for 5 min (repeated three times). Supernatants collected from two rounds of extraction were pooled after centrifugation (12,000 rpm, 15 min, 4 °C), dried with nitrogen gas, subjected to derivatization with methanol–(trimethylsilyl)diazomethane (1:2, *v*/*v*) at room temperature for 30 min, and finally taken up in n-hexane for injection. GC–MS was performed on a DB-Fast FAME column with helium as the carrier gas, with instrument parameters and temperature program set as described above, and the detector operated in SIM mode. By cross-referencing retention times and characteristic ions with reference standards, analytes were identified and quantified. For vitamin C determination, a separate aliquot of ovarian tissue was extracted with a cold metaphosphoric acid solution containing dithiothreitol, and analyzed by high-performance liquid chromatography with ultraviolet detection (HPLC-UV) at 265 nm following a validated protocol.

### 2.4. Quality Control, Library Preparation, and Sequencing

Ovary samples were transported on dry ice to Beijing Skyao Biomedical Technology Co., Ltd. (Beijing, China) for high-throughput sequencing. RNA extraction, quality assessment, reverse transcription, cDNA library construction, and sequencing were performed following Chen et al. [3]. Briefly, mRNA was enriched using Oligo(dT)-coupled magnetic beads, randomly fragmented, and reverse-transcribed into cDNA, followed by end repair, A-tailing, adapter ligation, size selection, and PCR amplification. Libraries were quantified (Qubit 3.0), size-verified (Qsep400), and qualified libraries (effective concentration > 2 nM) were sequenced on a high-throughput platform in PE150 mode.

### 2.5. De Novo Transcriptome Assembly and Annotation

Raw sequencing data were processed with CASAVA for base calling and converted to FASTQ format. Quality filtering was then performed to remove low-quality reads (Qphred ≤ 20), adapter-contaminated reads, and those with excessive poly-N content, yielding high-quality clean reads. The Q20, Q30, GC content, and duplication levels of clean reads were calculated for downstream analyses. Clean reads from each sample were assembled de novo using Trinity [21] to generate transcript sequences, and RSEM [22] was used to quantify gene expression levels. For functional annotation, assembled transcripts were searched against multiple public databases via BLASTX (version 2.15.0) with an E value cutoff of 1.0 × 10^−5^.

### 2.6. Differential Expression Analysis

The differential expression between Stage II and Stage IV ovarian samples was analyzed with the DESeq R package (version 2.0), which employs a negative binomial distribution model. The Benjamini–Hochberg procedure was applied to control the false discovery rate (FDR) and adjust raw *p*-values. Genes with adjusted *p*-values (padj) < 0.05 were considered differentially expressed. Identified differentially expressed genes (DEGs) were then functionally annotated against the GO and KEGG databases. GO enrichment analysis was conducted using the topGO R package (version 2.50.0) with the Kolmogorov–Smirnov test, and KEGG pathway enrichment analysis was performed using KOBAS software (version 3.0) [23]. Statistical significance for all enrichment analyses was defined as a padj value less than 0.05.

### 2.7. qPCR Analysis

To validate the transcriptome data, 9 DEGs were quantified by qPCR using primers designed with Primer Premier 6.0 (Table 1). Total RNA was extracted from ovarian tissues using Trizol-A+ (DP421, TianGen Biotech Co., Ltd., Beijing, China), and its integrity, purity, and concentration were assessed using an Agilent 2100 Bioanalyzer (Agilent Technologies, Santa Clara, CA, USA) and a NanoDrop 2000 spectrophotometer (Thermo Fisher Scientific, Waltham, MA, USA). After reverse transcription of qualified RNA with FastKing-RT SuperMix (KR118, TianGen, China), qPCR was conducted with 2× SYBR qPCR Mix (High ROX) (Aidlab Biotechnologies Co., Ltd., Beijing, China) on an AB StepOnePlus Real-Time PCR System (Applied Biosystems, Foster City, CA, USA), applying the conditions of 95 °C for 3 min and 35 cycles of 95 °C for 10 s plus 65 °C for 30 s. Each sample was run in triplicate, and relative expression levels were calculated using the 2^−ΔΔCT^ method with *eef1a1* (eukaryotic translation elongation factor 1 alpha 1) as the reference gene.

### 2.8. Statistical Analysis

All data were expressed as the mean ± standard error of the mean (SEM) of three replicates. Statistical comparisons were performed using an independent-samples *t*-test, with the significance level set at 0.05. Normality (one-sample Kolmogorov–Smirnov test) and homogeneity of variance (Levene’s test) were assessed prior to analysis. All statistical analyses were conducted using IBM SPSS Statistics 22.0 (IBM Corp., Armonk, NY, USA).

## 3. Results

### 3.1. Morphological Indices

Morphological parameters of LBG at ovarian Stages II and IV are presented in Table 2. Body weight, standard length, viscera weight, and gonad weight were significantly higher in G4 than in G2 fish (*p* < 0.05). Condition factor (CF) did not differ significantly between stages (*p* > 0.05). Viscerosomatic index increased markedly from 3.88% to 8.20%, and gonadosomatic index from 0.43% to 3.40% during the transition from Stage II to IV (*p* < 0.05).

### 3.2. Proximate Composition

Proximate composition of the whole fish is presented in Table 3. Moisture trended to decrease from 67.1% to 62.8% across ovarian stages II to IV, though the difference was not significant (*p* > 0.05). Crude protein percentage did not differ significantly between stages (*p* > 0.05). Both crude lipid and ash levels increased significantly with ovarian maturation from Stage II to IV (*p* < 0.05), rising from 14.9% to 17.4% and from 1.52% to 1.83%, respectively.

### 3.3. Target Metabolomics for Fatty Acid Profiles

**Figure 2:**
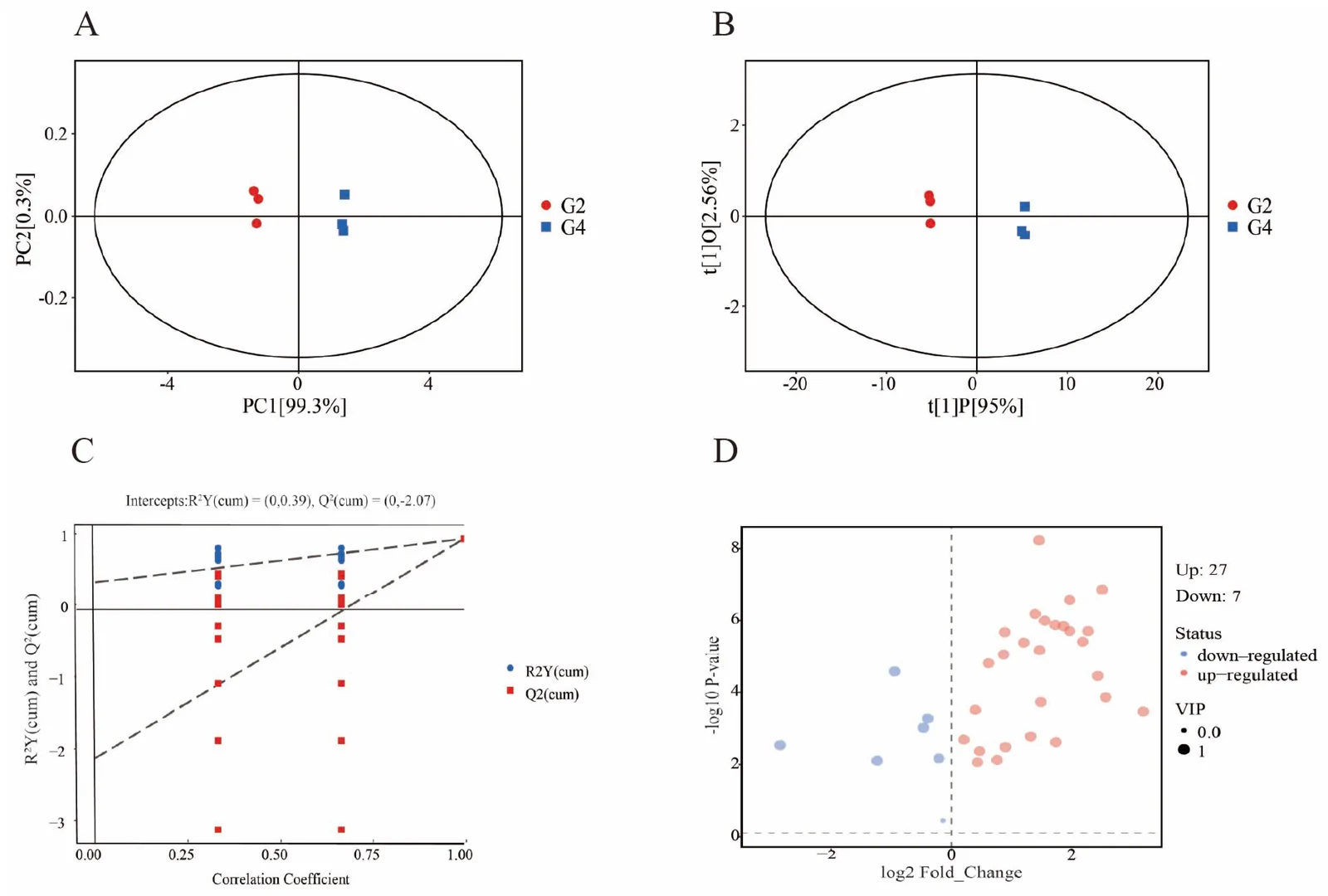
GC-MS-based profiling of fatty acids in largemouth bronze gudgeon (*C. guichenoti*) ovaries at Stages II and IV. (**A**) Principal component analysis. (**B**) OPLS-DA score scatter plot. (**C**) OPLS-DA permutation test plot. (**D**) Volcano plot. Differential metabolites were filtered using the following criteria: |log_2_FC| > 1, VIP > 1, and *p* < 0.05. Red and blue indicate upregulated and downregulated fatty acids, respectively.

GC–MS-based profiling of fatty acids in LBG ovaries at Stages II and IV is shown in Figure 2. Principal component analysis (PCA) revealed that PC1 and PC2 explained 99.0% and 0.3% of the total variance, respectively, yielding a cumulative contribution of 99.3% (Figure 2A). PCA also revealed clear separation of G2 and G4 samples into distinct clusters, indicating good repeatability within each group. The orthogonal partial least squares-discriminant analysis (OPLS-DA) score plot showed that G2 and G4 were well separated along the t[1]P predictive component without overlap, and that all samples resided within the 95% confidence ellipse (Figure 2B). Along the orthogonal component t[1]O, samples within each group clustered tightly, with no obvious outliers. A 200-time permutation test was applied to verify the OPLS-DA model (Figure 2C). Exhibiting a Q^2^ regression intercept of −2.07 (Q^2^ < 0), the permuted models yielded substantially lower Q^2^ values compared to the original model, which indicated the absence of overfitting and validated significant intergroup metabolic differences. Overall, distinct differences in fatty acid profiles existed between Stage II and IV ovaries, and the OPLS-DA model effectively discriminated between the two developmental stages, providing a reliable basis for subsequent differential metabolite screening. A total of 34 fatty acids were detected above the limit of quantification and identified as significant differential metabolites in G4 versus G2 ovaries, including 27 upregulated and 7 downregulated (Figure 2D). Full lists of differential fatty acids are provided in Supplementary Table S1.

### 3.4. Fatty Acid Composition

Fatty acid profiles in LBG ovaries are presented in Table 4. After excluding trans-, odd-chain fatty acids, and those with absolute levels below 5 μg/g (relative content < 0.12%), 19 fatty acids were identified. The six most abundant fatty acids, including docosahexaenoic acid (C22:6n-3, DHA), oleic acid (C18:1n-9), palmitic acid (C16:0), linoleic acid (C18:2n-6, LA), stearic acid (C18:0), and arachidonic acid (ARA, C20:4n-6), collectively accounted for approximately 80% of total fatty acids in both stages. C18:2n-6 predominated in G2, whereas DHA was the most abundant in G4 ovaries. Relative to G2, C16:0, C18:0, ARA, and DHA percentages in G4 ovaries increased by 29.3%, 51.5%, 28.4%, and 253%, respectively, while C18:1n-9 and C18:2n-6 significantly decreased by 27.6% and 55.2%, respectively (*p* < 0.05). By category, total SFA increased by 33.5%, MUFA decreased by 31.5%, and PUFA markedly increased by 9.7% (*p* < 0.05). Specifically, the n-3 PUFA percentage rose by 134%, whereas n-6 PUFA significantly declined by 33.1% (*p* < 0.05). Despite the reduction in total n-6 PUFA, ARA and its metabolism-related fatty acids (C20:2n-6, C20:3n-6, C22:4n-6, and C22:5n-6) were all significantly higher in G4 than in G2 ovaries (*p* < 0.05).

### 3.5. Vitamin Composition

The levels of fat-soluble vitamins and VC are shown in Table 5. A significant decrease in VA from 1101 to 595 ng/g was observed during ovarian development (Stages II–IV; *p* < 0.05), in contrast to the marked increases in VC and VE (*p* < 0.05). VD was below detection, and VK concentrations were similar between stages (*p* > 0.05). Due to the limited number of differentially expressed vitamins, no further multivariate analysis was performed.

### 3.6. Transcriptome Analysis

#### 3.6.1. Differential Expression Analysis

**Figure 3:**
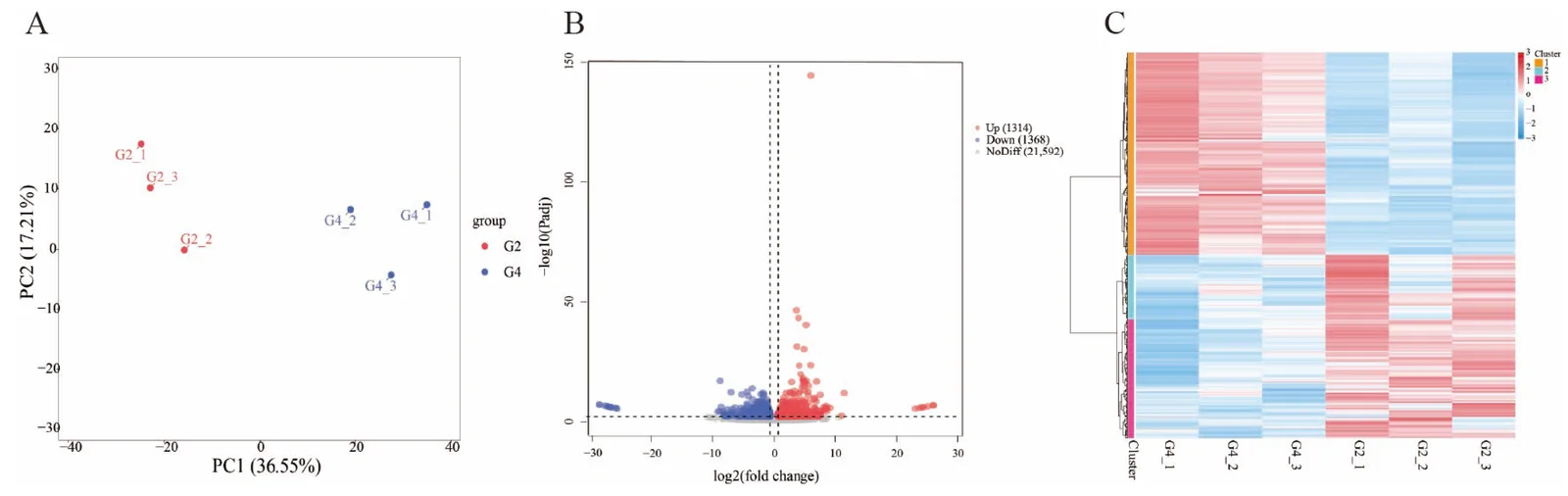
Principal component and differential expression analyses of the transcriptome. (**A**) Principal component analysis. (**B**) Volcano plot. (**C**) Cluster heatmap. G2, Stage II ovary. G4, Stage IV ovary.

PCA and differential expression analysis of transcriptome data are shown in Figure 3. PCA revealed clear intergroup separation and tight intragroup clustering of replicates. PC1 and PC2 explained 36.6% and 17.2% of the total variance, respectively. Although their combined contribution was 53.8%, the distinct clustering of samples from the two developmental stages indicates that ovarian stage is the primary factor driving transcriptional differences. The tight clustering of biological replicates within each group further supports the high reproducibility and reliability of the transcriptomic data (Figure 3A). Genes with |log_2_(FC)| ≥ 1.5 and adjusted *p* < 0.05 were defined as differentially expressed genes (DEGs). A total of 2682 DEGs were identified, with 1314 upregulated and 1368 downregulated (Figure 3B). The heatmap (Figure 3C) showed distinct bidirectional expression patterns between groups, with highly consistent profiles within each group, further confirming the reliability of intergroup differences. Full lists of DEGs are provided in Supplementary Table S2.

#### 3.6.2. GO Enrichment Analysis of DEGs

**Figure 4:**
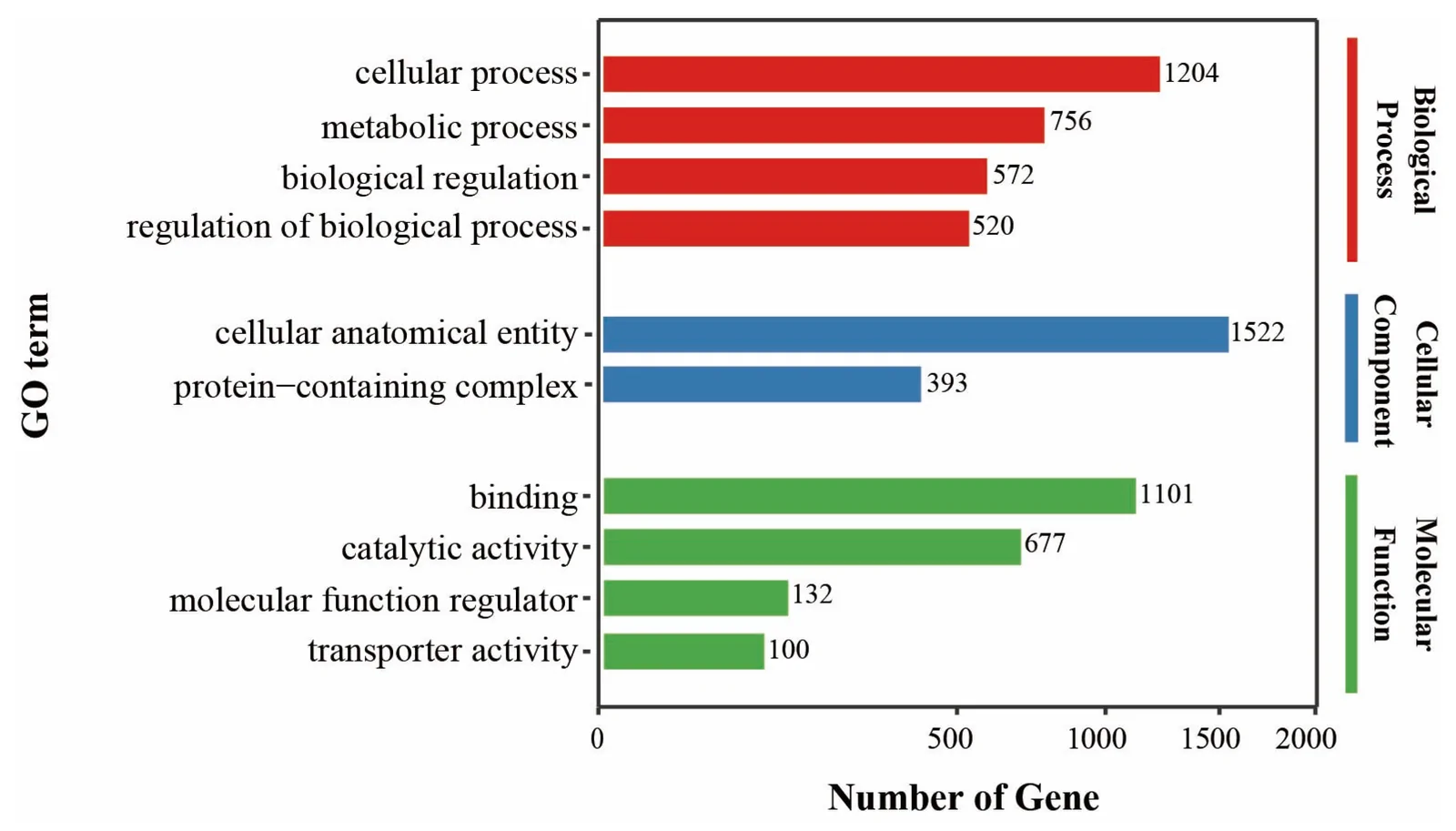
GO enrichment of DEGs in Stage IV ovaries versus Stage II ovaries of largemouth bronze gudgeon (*C. guichenoti*).

GO enrichment analysis classified DEGs into three main categories: Biological Process (BP), Cellular Component (CC), and Molecular Function (MF) (Figure 4). Compared with G2 ovaries, the enriched BP terms in G4 ovaries included cellular processes, metabolic processes, and biological regulation; CC terms included cellular anatomical entities and protein-containing complexes; and MF terms were dominated by binding and catalytic activity.

#### 3.6.3. KEGG Enrichment Analysis of DEGs

**Figure 5:**
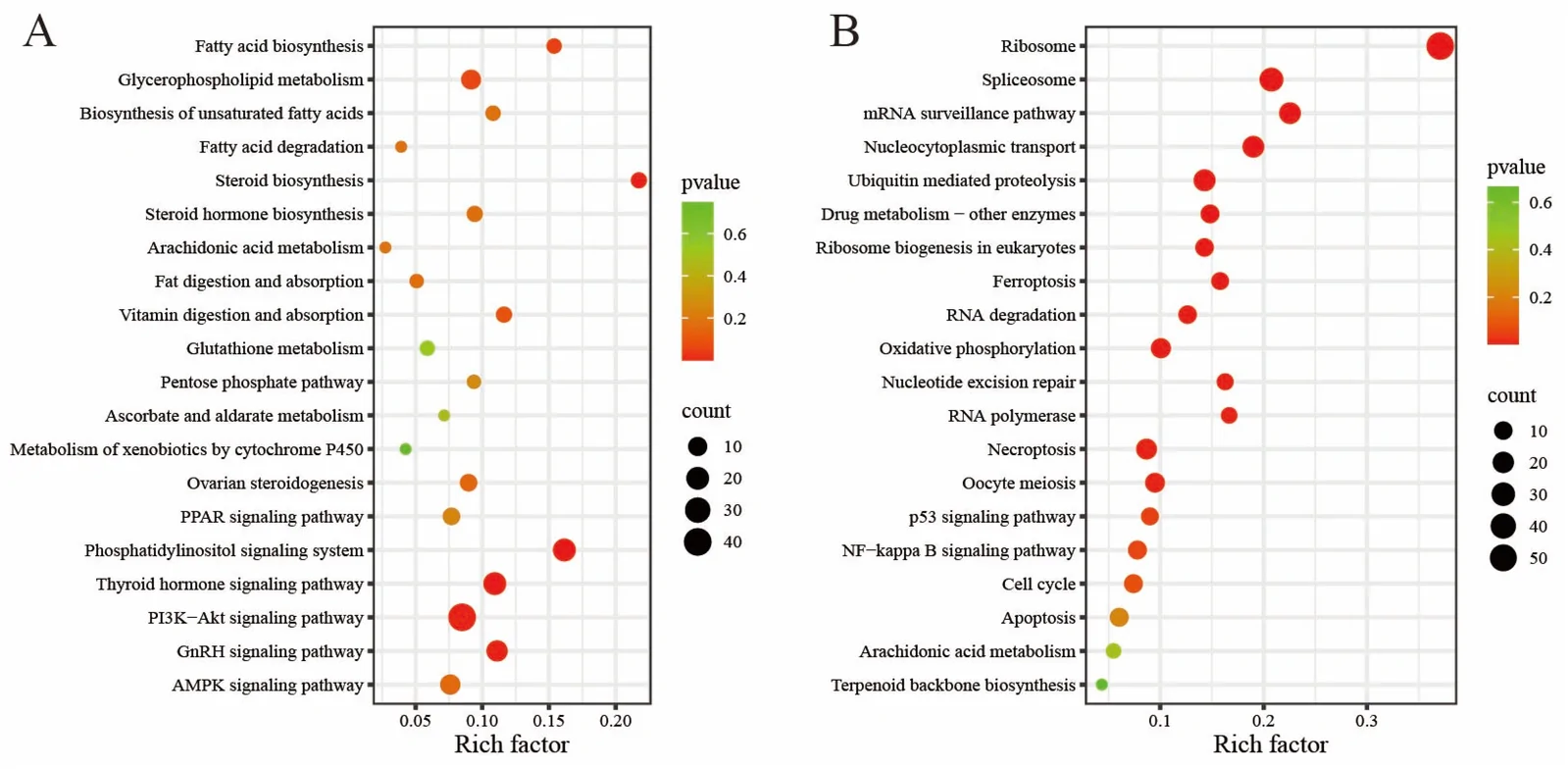
KEGG pathway enrichment of DEGs in Stage IV ovaries versus Stage II ovaries of largemouth bronze gudgeon (*C. guichenoti*). (**A**) Upregulated DEGs. (**B**) Downregulated DEGs.

KEGG enrichment analysis of DEGs is shown in Figure 5. The results showed that, relative to G2 ovaries, the upregulated DEGs in G4 ovaries were mainly enriched in the PI3K-Akt signaling pathway and lipid metabolism-related pathways, including fat digestion and absorption, fatty acid biosynthesis, unsaturated fatty acid biosynthesis, fatty acid degradation, glycerophospholipid metabolism, ARA metabolism, and the PPAR signaling pathway (Figure 5A). In addition, reproductive hormone-related pathways, including steroid (hormone) biosynthesis, ovarian steroidogenesis, and GnRH signaling, as well as vitamin digestion/absorption, were also significantly enriched. In contrast, downregulated DEGs showed enrichment mainly in pathways linked to cell proliferation and transcriptional control, including ribosome biogenesis, RNA processing, oxidative phosphorylation, oocyte meiosis, and cell cycle (Figure 5B).

**Figure 6:**
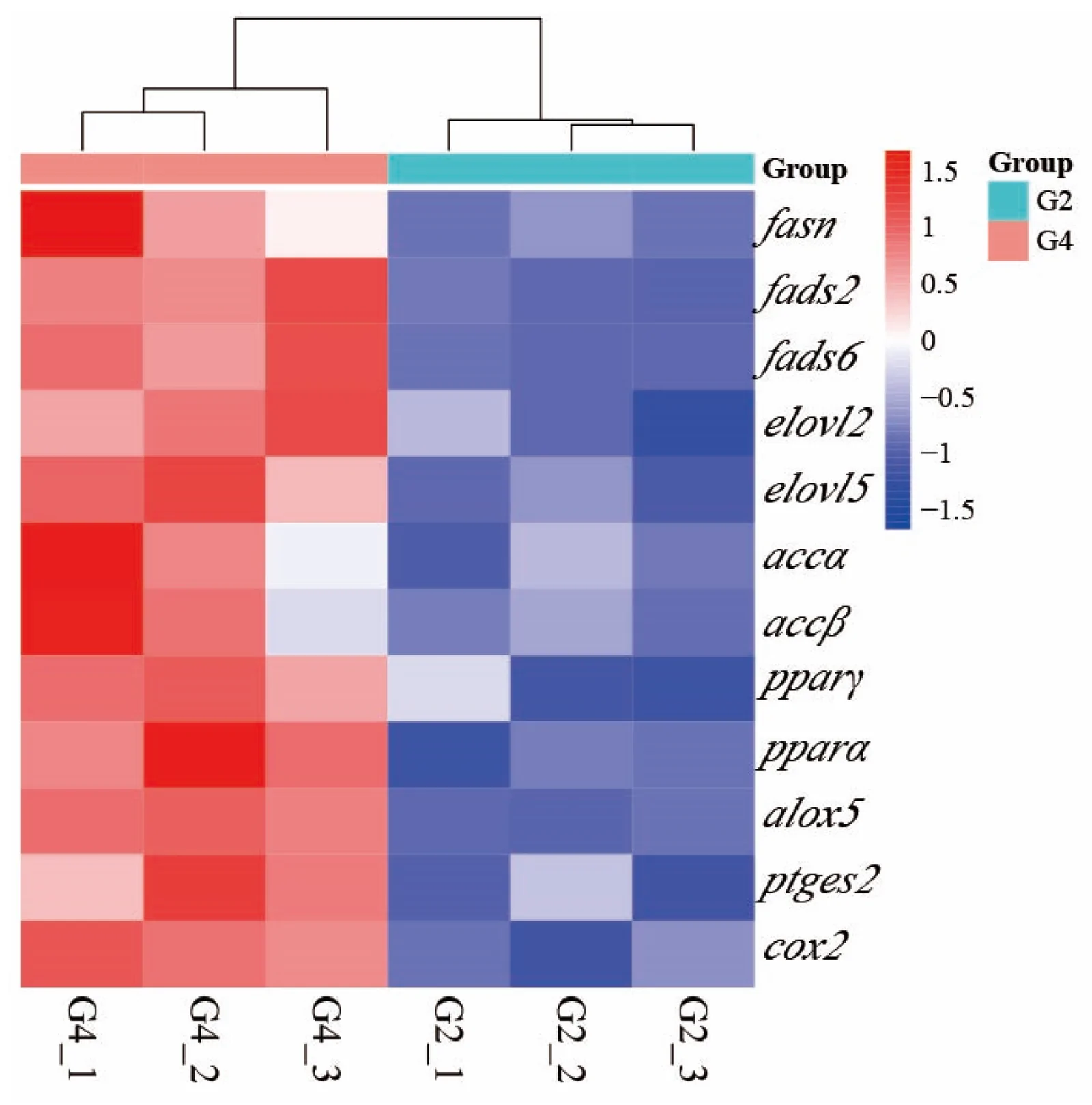
Clustering heatmaps of DEGs related to fatty acid metabolism in Stage IV ovaries versus Stage II ovaries of largemouth bronze gudgeon (*C. guichenoti*). *fasn*, fatty acid synthase; *fads*, fatty acid desaturase; *elovl*, fatty acid elongase; *acc*, acetyl-CoA carboxylase; *ppar*, peroxisome proliferator-activated receptor; *alox5*, arachidonate 5-lipoxygenase; *ptges2*, prostaglandin E synthase 2; *cox2*, cyclooxygenase 2.

To elucidate the effects of ovarian development from Stage II to IV on lipid metabolism, specific DEGs were extracted from the transcriptome (Figure 6). Genes involved in de novo fatty acid synthesis, including acetyl-CoA carboxylase alpha (*accα*), *accβ*, and fatty acid synthase (*fasn*), as well as those involved in unsaturated fatty acid synthesis, including fatty acid desaturase (*fads2* and *fads6*) and elongation of very long chain fatty acid protein (*elovl2* and *elovl5*), were significantly upregulated in G4 ovaries. In addition, the expression of arachidonate 5-lipoxygenase (*alox5*), prostaglandin E synthase 2 (*ptges2*), and cyclooxygenase 2 (*cox2*) was also markedly upregulated in G4 as compared with G2 ovaries.

### 3.7. Validation of DEGs by qPCR

**Figure 7:**
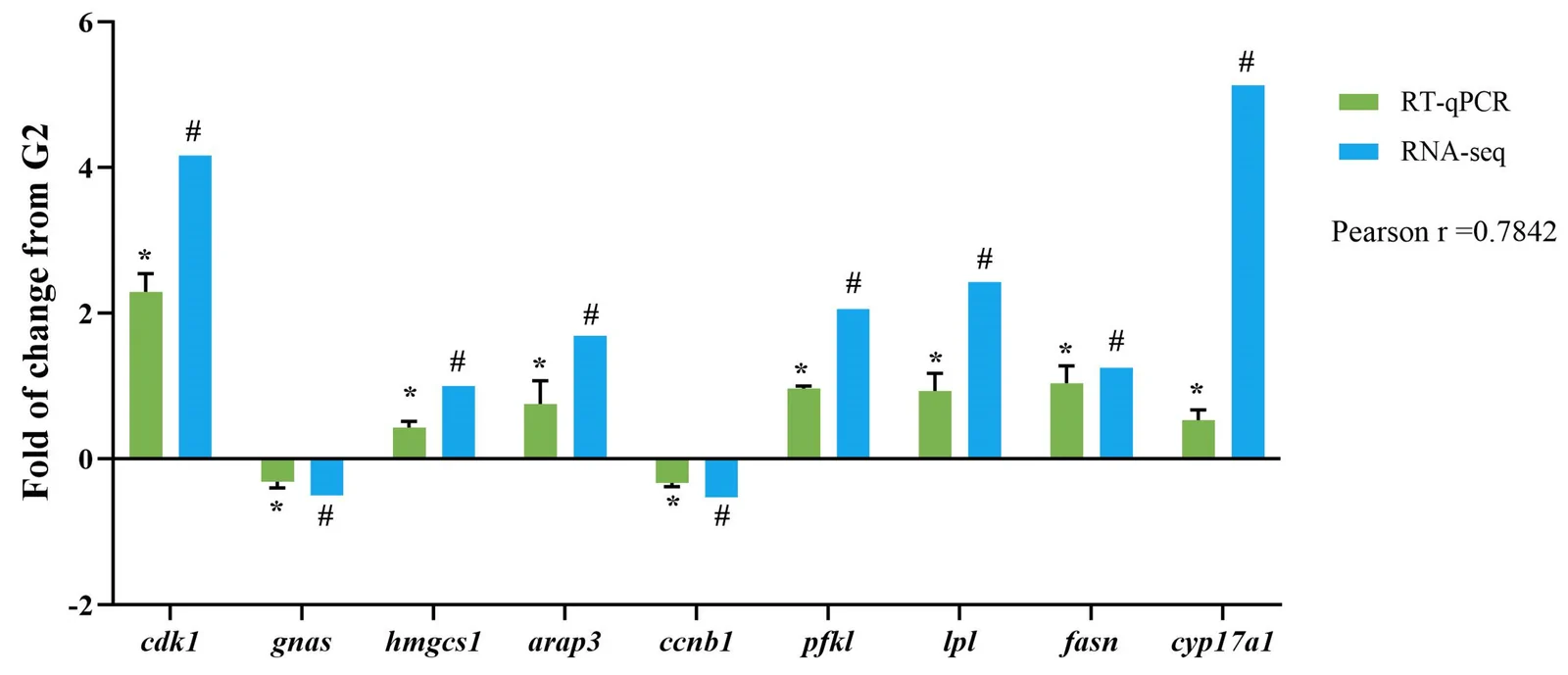
Validation of DEGs in Stage IV ovaries versus Stage II ovaries of largemouth bronze gudgeon (*C. guichenoti*) by real-time PCR analysis. For RT-qPCR, relative expression was normalized against *eef1a1*, values are means ± SEM (*n* = 3). An asterisk (*) on a column indicates a significant difference (*p* < 0.05). For RNA-seq, an asterisk (#) on a column indicates fold change (absolute values > 2.0) and FDR-corrected *p*-value (*p* < 0.05).

qPCR validation of RNA-seq data is presented in Figure 7. Relative to G2 ovaries, the mRNA levels of the GNAS complex locus (*gnas*) and cyclin B1 (*ccnb1*) were significantly downregulated in G4 ovaries (*p* < 0.05), while the other seven selected DEGs exhibited the opposite pattern. The expression profiles of all validated genes matched the RNA-seq data, confirming the reliability of the transcriptomic results.

## 4. Discussion

Vitellogenesis, the physiological process of yolk granule accumulation within oocytes, is the defining hallmark of ovarian progression from Stage II (previtellogenic) to Stage IV (late vitellogenic) in fish [7,24]. In many teleost species, yolk is a major lipid reservoir [25,26]. In this study, both viscerosomatic and gonadosomatic indices of LBG increased dramatically during the Stage II–IV transition, accompanied by a marked elevation in whole-body lipid content. Targeted metabolomics revealed that total ovarian fatty acid content rose from 4.15 mg/g in G2 to 10.7 mg/g in G4 (wet weight basis), suggesting that this developmental window is characterized by active lipid anabolism and mobilization. Specifically, total SFA increased from 20.1% to 26.8%, and total PUFA from 44.8% to 49.1%, across ovarian Stages II to IV. The rise in SFA was mainly attributable to C16:0 and C18:0, which are preferentially deposited as neutral lipids in the yolk to provide metabolic energy for subsequent embryogenesis [27]. In contrast, the marked PUFA accumulation is primarily directed toward rapid plasma and organelle membrane expansion during oocyte maturation [28,29]. Collectively, the pronounced increases in SFA and PUFA reflect the physiological demands of energy storage and structural construction during late ovarian development in LBG and constitute the material prerequisite for yolk deposition and oocyte enlargement.

As a key structural element of membrane phospholipids, DHA directly ensures the proper development of oocytes and the neural and visual systems in larval fish [11,30,31]. In this study, DHA was responsible for the pronounced increase in total n-3 PUFA, which rose dramatically from 6.89% in G2 to 24.3% in G4 ovaries, suggesting an efficient DHA enrichment mechanism during this developmental window in LBG. Although EPA is generally considered vital for egg development in fish [32,33], its role in vitellogenesis of LBG appeared limited, given its low concentration (less than 1.64%). In contrast to total PUFA, n-6 PUFA decreased from 33.3% to 22.3%, primarily due to a sharp decline in LA (C18:2n-6). Despite the reduction in n-6 PUFA, ARA increased from 4.29% to 5.50%, along with its metabolism-related fatty acids (C20:2n-6, C20:3n-6, C22:4n-6, and C22:5n-6), all of which were elevated during this developmental window. These results suggested that ARA might play a critical physiological role in the reproductive regulation of LBG. In addition to these marked changes in DHA and ARA, other fatty acids also exhibited notable alterations. The contents of oleic acid (C18:1n-9) and LA decreased, contrasting with many studies reporting increases in these fatty acids during ovarian development in fish [34,35]. This discrepancy may indicate their preferential utilization either for energy production or as substrates for long-chain PUFA synthesis [25,36]. Meanwhile, EPA content declined slightly, suggesting that oocytes of *C. guichenoti* preferentially accumulate DHA over EPA during development, a pattern consistent with the fatty acid allocation characteristics of river-resident fish species [37,38].

To further elucidate the mechanisms underlying fatty acid profile changes during ovarian maturation in LBG, transcriptomic analysis was performed in this study. As expected, many upregulated DEGs in G4 ovaries were enriched in lipid metabolism pathways. Specifically, elevated C16:0 and C18:0 levels were consistent with upregulated expression of genes involved in de novo fatty acid synthesis (*accα*, *accαβ*, and *fasn*). Similarly, increased DHA, ARA, and their metabolism-related fatty acids corresponded with upregulated desaturases (*fads2* and *fads6*) and elongases (*elovl2* and *elovl5*). In fish, PPARα serves as a key nuclear regulator of lipolysis, enhancing mitochondrial fatty acid β-oxidation upon activation [39,40]. In this study, upregulated *pparα* expression in G4 ovaries may account for reduced levels of certain fatty acids, such as C18:1n-9 and LA. In line with elevated ARA levels, the expression of arachidonate 5-lipoxygenase (*alox5*), prostaglandin E synthase 2 (*ptges2*), and cyclooxygenase 2 (*cox2*) was upregulated in G4 ovaries. These results support the notion that ARA participates in reproductive regulation primarily through the prostaglandin E_2_ (PGE_2_) signaling axis via the COX pathway in fish [41,42]. Consistent with our findings, Xia et al. [43] reported that the ARA and PG synthesis pathways are synchronously activated with ovarian maturation in wild female LBG.

In many fish species, exogenous ARA supplementation upregulates the expression of steroidogenic genes, including cytochrome P450 family 19 subfamily a member 1a (*cyp19a1a*) and steroidogenic acute regulatory protein (*star*), and promotes the secretion of follicular estrogen [18,44,45,46]. Apart from its structural role, DHA upregulates *cyp11a1*, leading to increased pregnenolone production and consequently driving oocyte maturation in zebrafish [15]. In line with elevated ARA and DHA levels, our transcriptomic data revealed that the steroid hormone biosynthesis pathway was enriched in G4 ovaries, with key genes including *cyp11a1*, *cyp17a1,* and *cyp19a1a* sequentially upregulated, forming a complete synthetic cascade from cholesterol to estradiol. These findings suggest that, beyond their structural roles, DHA and ARA may act as critical nutritional signaling molecules that facilitate ovarian maturation in LBG, likely through the activation of the hypothalamic-pituitary-gonadal (HPG) axis. Additionally, many upregulated DEGs in G4 ovaries were enriched in GnRH and thyroid hormone signaling pathways, supporting the involvement of these hormones in fish reproduction [47,48]. From a practical standpoint, these results imply that dietary supplementation with a balanced ratio of DHA and ARA, rather than merely high levels of total n-3 PUFAs, should be a focal point in developing effective broodstock feeds for LBG.

In contrast, the downregulated DEGs were mainly enriched in pathways related to cell proliferation and transcriptional control, including ribosome biogenesis, RNA processing, the cell cycle, and oocyte meiosis. In Stage II, active proliferation sustains high expression of ribosome and RNA splicing genes [49,50], whereas Stage IV shows reduced transcriptional demands [51,52]. Suppression of ribosome and RNA processing pathways reflects a reprogramming that shifts priority from cell division to yolk lipid deposition [53]. De novo transcription declines, while maternal mRNA stability and translation become crucial for meiosis resumption and early embryogenesis [54]. This pattern is consistent with the conserved transcriptional remodeling during late vitellogenesis in other teleosts [55,56].

Many studies have demonstrated that dietary supplementation with VC or VE, alone or in combination, can improve ovarian development in fish [57,58,59]. An elevation in VC and VE levels across ovarian Stage II to Stage IV was revealed by targeted metabolomics in this study, indicating that these vitamins play crucial roles in the maturation of LBG ovaries. Consistently, ascorbate and aldarate metabolism, along with the vitamin digestion and absorption pathway, were enriched in G4 ovaries based on transcriptomic data. Specifically, multiple B vitamin transporter genes, including *slc52a3* (riboflavin), *slc5a6* (biotin and pantothenic acid), *slc19a1* (folate), and ATP-binding cassette subfamily C member 1 (*abcc1*, cobalamin), were upregulated [60,61,62,63,64], implying active transport of various B vitamins into the ovary to meet developmental demands in LBG. This warrants attention in the nutrient fortification of broodstock diets for LBG in the future.

The integrated metabolomic and transcriptomic approach validated the feasibility of identifying key nutrients using microscale tissue samples, which is especially valuable for rare species subject to destructive sampling. The key nutrients identified, including DHA, ARA, VC, and VE, can inform quantitative supplementation standards for LBG broodstock diets. Targeted supplementation is expected to overcome ovarian maturation arrest, improve egg quality and fertilization rates, and ultimately support stock enhancement and release programs.

## 5. Conclusions

The overall results of this study suggest that DHA, ARA, VC, and VE are potential key nutrients regulating ovarian maturation in LBG, as revealed by targeted metabolomics and transcriptomic analyses. Transcriptome data also implied the importance of several B vitamins. Targeted fortification of these nutrients in broodstock diets holds strong promise for effectively improving the reproductive performance of LBG.

## Figures and Tables

**Table 1:** Primer sequences used for real-time PCR.

Genes	Forward Primer (5′-3′)	Reverse Primer (5′-3′)	Size (bp)	Tm
*eef1a1*	TGGGTGTTGGACAAACTGAA	CAACACCACCAGCAACAATC	190	60 °C
*pfkl*	AGACTGCAGAAAGGGCAAAA	TTCTCTGCGGAAGGTCTTGT	154	60 °C
*lpl*	AGTGTCAGTGAACCGTCGTAGC	CGCAGTTTCCTCCTAATCTCCT	159	60 °C
*fasn*	CTTCTGGCAAACGTCTTTCAC	GTGTTTTGTTCCCAGAGTGGA	234	60 °C
*cyp17a1*	TACCTCGTCCACAATCCA	GTTCTTCCATTCCTTCTCATC	276	60 °C
*ccnb1*	GAGGAAGAAGGAGGTCAAG	ATCAAGCAGAACATCAGAGA	202	60 °C
*cdk1*	CTGGACAAGAACGGCATT	TGAGGTTACTGGCTGGAA	283	60 °C
*gnas*	TGTCAATGGCTTCAATGCT	GCTAACTGGACTGGAGGTA	123	60 °C
*hmgcs1*	TGGCTCTGCTCTGGATAA	CATACTGTCTGCGATGCT	218	60 °C
*arap3*	GAGACTGGTGGACTGTTAC	GGAGGACTTGGAGGTGAA	185	60 °C

*pfkl*: phosphofructokinase, liver type; *lpl*: lipoprotein lipase; *fasn*: fatty acid synthase; *itga2*: integrin subunit alpha 2; *cyp17a1*: cytochrome P450, family 17, subfamily A, polypeptide 1; *ccnb1*: cyclin B1; *cdk1*: cyclin dependent kinase 1; *gnas*: GNAS complex locus; *hmgcs1*: HMGCS13-hydroxy-3-methylglutaryl-CoA synthase 1; *arap3*: ARAP3ArfGAP with RhoGAP domain, ankyrin repeat and PH domain3. *eef1a1*, eukaryotic translation elongation factor 1 alpha 1.

**Table 2:** Morphological parameters of largemouth bronze gudgeon (*C. guichenoti*) at ovarian Stages II and IV.

Treatments	G2	G4
Body weight (g)	287 ± 11 ^b^	619 ± 23 ^a^
Standard length (cm)	26.4 ± 0.1 ^b^	33.2 ± 0.8 ^a^
Visceral mass (g)	10.7 ± 1.0 ^b^	50.0 ± 0.2 ^a^
Ovary weight (g)	1.23 ± 0.05 ^b^	20.6 ± 2.2 ^a^
Condition factor (g/cm^3^)	1.55 ± 0.08	1.69 ± 0.07
Viscerosomatic index (%)	3.88 ± 0.48 ^b^	8.20 ± 0.32 ^a^
Gonadosomatic index (%)	0.43 ± 0.01 ^b^	3.40 ± 0.50 ^a^

Values are means ± SEM (*n* = 3). Different superscript letters on the same row of parameters indicate a significant difference (*p* < 0.05). Condition factor = body weight/body length^3^ × 100. Viscerosomatic index = (Viscera weight/body weight) × 100. Gonadosomatic index = (Gonad weight/body weight) × 100.

**Table 3:** Proximate composition (% dry matter basis) in the whole body of largemouth bronze gudgeon (*C. guichenoti*) at ovarian Stages II and IV.

Treatments	G2	G4
Moisture (%)	67.1 ± 1.1	62.8 ± 2.5
Crude protein (%)	16.7 ± 0.4	16.8 ± 0.8
Crude lipid (%)	14.9 ± 0.5 ^b^	17.4 ± 1.0 ^a^
Ash (%)	1.52 ± 0.06 ^b^	1.83 ± 0.08 ^a^

Values are means ± SEM (*n* = 3). Different superscript letters on the same row of parameters indicate a significant difference (*p* < 0.05).

**Table 4:** Fatty acid composition (% of total fatty acids) in the ovaries of largemouth bronze gudgeon (*C. guichenoti*) at Stages II and IV.

Fatty Acid (%)	G2	G4
C14:0	0.72 ± 0.01 ^a^	0.37 ± 0.00 ^b^
C16:0	13.1 ± 0.10 ^b^	16.9 ± 0.3 ^a^
C18:0	6.31 ±0.07 ^b^	9.56 ± 0.05 ^a^
ΣSFA	20.1 ± 0.0 ^b^	26.8 ± 0.2 ^a^
C16:1n-7	6.15 ± 0.09 ^a^	2.07 ± 0.01 ^b^
C18:1n-7	2.39 ± 0.03	2.56 ± 0.05
C18:1n-9	26.1 ± 0.3 ^a^	18.9 ± 0.1 ^b^
C20:1n-9	0.50 ± 0.00 ^b^	0.57 ± 0.01 ^a^
ΣMUFA	35.1 ± 0.1 ^a^	24.1 ± 0.1 ^b^
C18:2n-6/LA	26.3 ± 0.0 ^a^	11.8 ± 0.1 ^b^
C18:3n-6	0.14 ± 0.00 ^a^	0.12 ± 0.00 ^b^
C20:2n-6	1.13 ± 0.01 ^b^	1.71 ± 0.03 ^a^
C20:3n-6	0.83 ± 0.00 ^b^	1.90 ± 0.02 ^a^
C20:4n-6/ARA	4.29 ± 0.03 ^b^	5.50 ± 0.04 ^a^
C22:4n-6	0.22 ± 0.00 ^b^	0.46 ± 0.01 ^a^
C22:5n-6	0.36 ± 0.01 ^b^	0.79 ± 0.01 ^a^
C18:3n-3	2.28 ± 0.03 ^a^	0.67 ± 0.01 ^b^
C20:3n-3	0.16 ± 0.00	0.17 ± 0.00
C20:5n-3/EPA	1.64 ± 0.00 ^a^	1.18 ± 0.02 ^b^
C22:5n-3	0.52 ± 0.00	0.53 ± 0.01
C22:6n-3/DHA	6.89 ± 0.05 ^b^	24.3 ± 0.1 ^a^
ΣPUFA	44.8 ± 0.2 ^b^	49.1 ± 0.3 ^a^
Σn-6PUFA	33.3 ± 0.1 ^a^	22.3 ± 0.2 ^b^
Σn-3PUFA	11.5 ± 0.1 ^b^	26.8 ± 0.2 ^a^

Values are means ± SEM (*n* = 3). ΣSFA, total saturated fatty acids; ΣMUFA, total monounsaturated fatty acids; ΣPUFA, total polyunsaturated fatty acids; Σn-6 PUFA, total n-6 polyunsaturated fatty acids; Σn-3 PUFA, total n-3 polyunsaturated fatty acids. Different superscript letters on the same row of parameters indicate a significant difference (*p* < 0.05).

**Table 5:** Vitamin content (ng/g wet weight) in the ovaries of largemouth bronze gudgeon (*C. guichenoti*) at Stages II and IV.

Vitamin (ng/g)	G2	G4
Retinol (VA)	1101 ± 59 ^a^	595 ± 29 ^b^
Ascorbic acid (VC)	11578 ± 305 ^b^	27888 ± 71 ^a^
α-Tocopherol (VE)	7845 ± 62 ^b^	23691 ± 54 ^a^
Phytonadione (VK1)	0.83 ± 0.08	0.78 ± 0.22

Values are means ± SEM (*n* = 3). Different superscript letters on the same row of parameters indicate a significant difference (*p* < 0.05).

## Data Availability

Raw sequence data are deposited at NCBI with bioproject accession number PRJNA1497645. The other data are available upon reasonable request.

## Supplementary Materials

The following supporting information can be downloaded at: https://www.mdpi.com/article/10.3390/ani16162602/s1.

## Abbreviations

ARA, Arachidonic acid; bp, Base pair; DHA, Docosahexaenoic acid; DEGs, Differentially expressed genes; FDR, False discovery rate; GC-MS, Gas chromatography-mass spectrometry; GO, Gene Ontology; GSI, Gonadosomatic index; KEGG, Kyoto Encyclopedia of Genes and Genomes; LA, Linoleic acid; LBG, Largemouth bronze gudgeon; MUFA, Monounsaturated fatty acid; OPLS-DA, Orthogonal partial least squares-discriminant analysis; PCA, Principal component analysis; PUFA, Polyunsaturated fatty acid; qPCR, Real-time quantitative PCR; SFA, Saturated fatty acid; VA, Vitamin A; VC, Vitamin C; VE, Vitamin E; VSI, Viscerosomatic index.
